# Zoonotic *Baylisascaris procyonis* Roundworms in Raccoons, China

**DOI:** 10.3201/eid2012.140970

**Published:** 2014-12

**Authors:** Yue Xie, Xuan Zhou, Mei Li, Tianyu Liu, Xiaobin Gu, Tao Wang, Weimin Lai, Xuerong Peng, Guangyou Yang

**Affiliations:** Sichuan Agricultural University, Ya’an, Sichuan, China

**Keywords:** Raccoon, roundworm, *Baylisascaris procyonis*, helminth, zoonosis, *Procyon lotor*, health education, pica, geophagia, China, parasites

**To the Editor:** *Baylisascaris procyonis*, an intestinal roundworm that infects raccoons (*Procyon lotor*), causes fatal or severe neural larva migrans in animals and humans (*1*,*2*). Globally, ≈130 species of wild and domesticated animals are susceptible (*2*). Infections in humans typically occur in children who have the disorders pica or geophagia and ingest *B*. *procyonis* eggs in items contaminated with raccoon feces (*3*). Clinical manifestations include ocular disease, eosinophilic encephalitis, and eosinophilic cardiac pseudotumors; severe infection can lead to death. Since 1984, ≈24 cases of *B. procyonis–*related human neural larva migrans have been reported, mainly in the United States (*1*,*3–5*; K.R. Kazacos, pers. comm.). Despite few cases among humans, lack of effective treatment and widespread distribution of infected raccoons in close association with humans make *B. procyonis* a potentially serious public health threat (*2*,*6*). The current distribution of *B. procyonis* is poorly recorded in Asia (*2*,*7*), except for Japan (*8*). We describe *B. procyonis* infections among raccoons in China as part of a series of ongoing surveys of helminthic zoonoses linked to captive exotic animals in zoologic gardens (ZGs) in China.

More than 90% of raccoons in China (n >320) are raised as exotic ornamental animals in 18 ZGs. During 2011–2013, we collected 2×308 fecal samples (i.e., 1 repeat within each sampling) from 277 raccoons in 12 randomly selected ZGs (Technical Appendix Figure 1). Samples were stored in individual plastic bags at –20°C until use. We examined raccoons (n = 31) at the Sichuan ZGs twice, in June 2012 and May 2013. We identified *B. procyonis* eggs in feces using morphologic and molecular analyses (*1*,*2*,*9*). The nuclear first internal transcribed spacer (428 bp) and mitochondrial cytochrome c oxidase subunit 1 (*cox-1*, 938 bp) genes in each sample were PCR-amplified and sequenced. *B. procyonis* infection was confirmed by sequencing and phylogenetic analyses of both genes (*7*,*9*). We reexamined ≈60% of fecal samples to validate results. Prevalence (95% CI) was calculated for the overall population and independently for female, male, juvenile, and adult raccoons. We determined differences between the tested ZG prevalence and prevalence by sex or age of raccoons using χ^2^ or Fisher exact tests in SAS (SAS Institute, Cary, NC, USA); p values <0.05 were considered significant.

Building on egg-based morphologic characterization and internal transcribed spacer 1 and *cox-1* gene-based phylogenies using neighbor-joining trees (Technical Appendix Figure 2), we found *B. procyonis* in raccoon feces from 5/12 ZGs (42%; 95% CI 14%–70%), including 2 in the most densely populated provinces, Henan and Sichuan. More infections were found in western than central and eastern ZGs (4/6 and 1/6, respectively; Table, Technical Appendix Figure 1) (p = 0.079). Fecal samples of 35 raccoons (13%; 95% CI 9%–17%) tested positive for *B. procyonis*. The mean intensity of egg shedding was 5,000 eggs per gram (range 800–11,200 eggs per gram; data not shown). No significant difference was observed in the intensity of shedding by comparing sex and age of animals, and no significant differences were noted in the mean prevalence between female and male raccoons (12% versus 14%; p = 0.677) or between adult and juvenile animals (13% versus 10%; p = 0.536).

**Table:** Prevalence of *B*aylisascaris *procyonis* roundworm infections among captive raccoons, China, 2011–2013*

Location, zoological gardens	No. *B. procyonis*–positive samples/total no. samples (%)						
	Sex				Age group		Total
	M		F		Adult	Juvenile	
Western region							33/146 (23)
Chongqing		–	0/4		0/4	–	0/4
Bifengxia		0/8	0/14		0/13	0/9	0/22, 0/22
Chengdu		0/4	1/5 (20)		0/6	1/3 (33)	1/9 (11), 0/9
Xi'an Wildlife		0/9	1/27 (4)		0/28	1/8 (13)	1/36 (3)
Kunming		12/12 (100)	15/15 (100)		22/22 (100)	5/5 (100)	27/27 (100)
Kunming Wildlife		1/24 (4)	3/24 (13)		4/48 (8)	–	4/48 (8)
Central region							2/56 (4)
Harbin Northern Forest		0/12	0/18		0/21	0/9	0/30
Zhengzhou		1/5 (20)	1/11 (9)		2/12 (17)	0/4	2/16 (13)
Changsha		0/3	0/7		0/5	0/5	0/10
Eastern region							0/75
Beijing		0/16	0/24		0/22	0/18	0/40
Guangzhou		0/5	0/15		0/20	–	0/20
Shanghai Wildlife		0/4	0/11		0/9	0/6	0/15
Total		14/102 (14)	21/175 (12)		28/210 (13)	7/67 (10)	35/277 (13)
p value		0.677			0.536		–

*Raccoons, considered to be exotic ornamental animals, are mainly kept in 18 zoologic gardens (ZGs) in China; 12 ZGs were examined for *B. procyonis *prevalence during the study period. Sichuan ZGs, including Bifengxia ZG and Chengdu ZG, were tested twice during this surveillance period. –, no raccoons in the group or no data available.

This investigation documents the presence and prevalence of *B. procyonis* among raccoons in China. The findings imply that raccoons harboring this parasite have the potential for spreading it to humans. One reason is that captive raccoons adapt readily to humans and easily take food offered by hand; another is that communal raccoon latrine sites in ZGs are usually close to areas where humans gather, so ZG visitors may be exposed to large numbers of eggs (Technical Appendix Figure 3). These eggs can remain viable and infective for years (*2*), and latrines are recognized as primary sources of transmission of *B. procyonis* to humans (*4*). Current public health initiatives to prevent *B. procyonis* infections in humans rely on the education of veterinary and human health care professionals, who in turn inform the public (*1*,*6*,*10*). Thus, veterinarians, clinicians, and public health officials in China should be more informed about this pathogen, especially in regions with large raccoon populations. 

Because of a lack of clinical awareness of this illness and subsequent lack of early diagnosis and effective treatment, prevention of *B. procyonis* infection by education is essential. In addition, a strategy for eradication is needed. Heat, in the form of boiling water, steam-cleaning, or fire, is the optimal tool for killing *B. procyonis* eggs (*2*) and therefore can be used to decontaminate areas surrounding latrines. Within heavily contaminated areas, removing and then sterilizing the top few inches of surface soil with heat would be effective and practical (*1*,*2*). Among captive raccoon populations, particularly in China, regular deworming is also likely to be helpful in reducing novel and existing sources of infection (*1*–*3*). 

Finally, although no cases of human infection have been reported in China to our knowledge, physicians should consider including *B. procyonis* infections in their differential diagnoses of patients with indicative features: clinical (eosinophilic encephalitis, ocular disease), epidemiologic (raccoon exposure), radiologic (white matter disease), and laboratory results (blood and CNS eosinophilia) (*1*,*10*). This study lays the foundation for future steps to educate the population of China about *B. procyonis* infection and to create programs to prevent the spread of this disease to humans.

Technical Appendix Distribution of raccoons in China, morphological and molecular characterization of *Baylisascaris procyonis* parasitic roundworm eggs in captive raccoons in China, and the potential risk of human *B. procyonis* infection in China.

## References

[1] Gavin PJ, Kazacos KR, Shulman ST. Baylisascariasis. Clin Microbiol Rev. 2005;18:703–18. 10.1128/CMR.18.4.703-718.2005 16223954 PMC1265913

[2] Kazacos K. *Baylisascaris procyonis* and related species. In: Samuel W, Pybus M, Kocan A, editors. Parasitic diseases of wild mammals. 2nd ed. Ames (IA): Iowa State University Press; 2001. p. 301–41.

[3] Murray WJ, Kazacos KR. Raccoon roundworm encephalitis. Clin Infect Dis. 2004;39:1484–92. 10.1086/425364 15546085

[4] Page LK, Anchor C, Luy E, Kron S, Larson G, Madsen L, Backyard raccoon latrines and risk for *Baylisascaris procyonis* transmission to humans. Emerg Infect Dis. 2009;15:1530–1. 10.3201/eid1509.090128 19788835 PMC2819851

[5] Kelly TG, Madhavan VL, Peters JM, Kazacos KR, Silvera VM. Spinal cord involvement in a child with raccoon roundworm (*Baylisascaris procyonis*) meningoencephalitis. Pediatr Radiol. 2012;42:369–73. 10.1007/s00247-011-2151-y 21629989

[6] Wise ME, Sorvillo FJ, Shafir SC, Ash LR, Berlin OG. Severe and fatal central nervous system disease in humans caused by *Baylisascaris procyonis*, the common roundworm of raccoons: a review of current literature. Microbes Infect. 2005;7:317–23 . 10.1016/j.micinf.2004.12.005 15715975

[7] Blizzard EL, Yabsley MJ, Beck MF, Harsch S. Geographic expansion of *Baylisascaris procyonis* roundworms, Florida, USA. Emerg Infect Dis. 2010;16:1803–4. 10.3201/eid1611.100549 21029553 PMC3294519

[8] Miyashita M. Prevalence of *Baylisascaris procyonis* in raccoons in Japan and experimental infections of the worm in laboratory animals. Journal of Urban Living and Health Association. 1993;37:137–51.

[9] Xie Y, Zhang Z, Niu L, Wang Q, Wang C, Lan J, The mitochondrial genome of *Baylisascaris procyonis.* PLoS ONE. 2011;6:e27066. 10.1371/journal.pone.0027066 22046447 PMC3203944

[10] Sorvillo F, Ash LR, Berlin OGW, Tatabe J, Degiorgio C, Morse SA. *Baylisascaris procyonis*: an emerging helminthic zoonosis. Emerg Infect Dis. 2002;8:355–9. 10.3201/eid0804.010273 11971766 PMC2730233

