# Supplementary material for: Zoonotic Baylisascaris procyonis Roundworms in Raccoons, China
Source: Emerg Infect Dis. 2014 Dec;20(12):2170–2. doi: 10.3201/eid2012.140970 (PMC4257832; doi:10.3201/eid2012.140970)
Supplement: Technical Appendix — Distribution of raccoons in China, morphological and molecular characterization of Baylisascaris procyonis parasitic roundworm eggs in captive raccoons in China, and the potential risk of human B. procyonis infection in China. [file 14-0970-Techapp-s1.pdf]

DOI: http://dx.doi.org/10.3201/eid2012.140970

Zoonotic *Baylisascaris procyonis* Roundworms in Raccoons, China

Technical Appendix

Distribution of raccoons in China, morphological and molecular characterization of *Baylisascaris procyonis* parasitic roundworm eggs in captive raccoons in China, and the potential risk of human infection with *B. procyonis* in China.

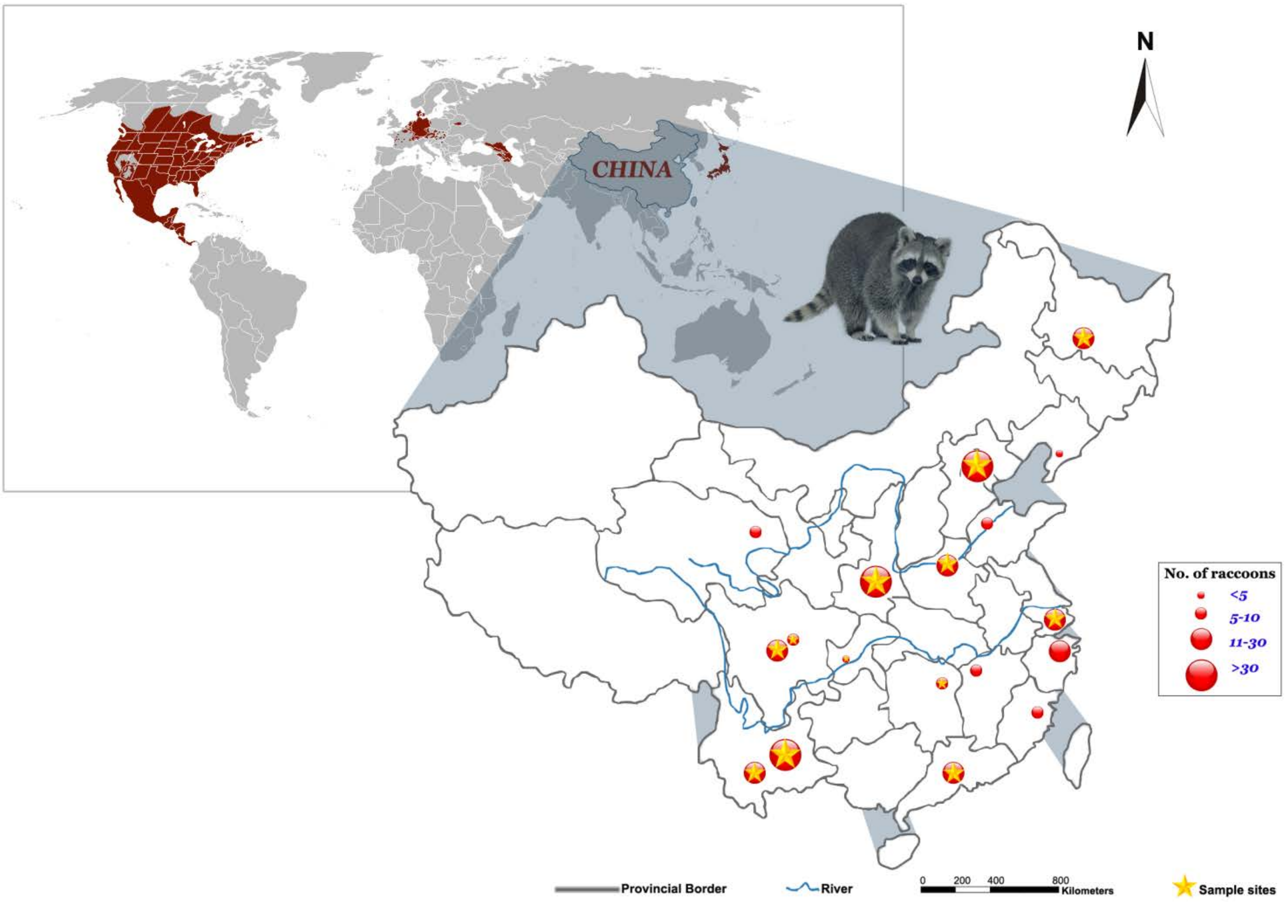

Technical Appendix Figure 1. Distribution of raccoons in China, 2011–2013. As exotic ornamental animals, >320 raccoons are kept in 18 Zoological Gardens (ZGs) in China. The size of the red circles represents the density of raccoons in each ZG during the study period. Sampling sites are marked with yellow stars.

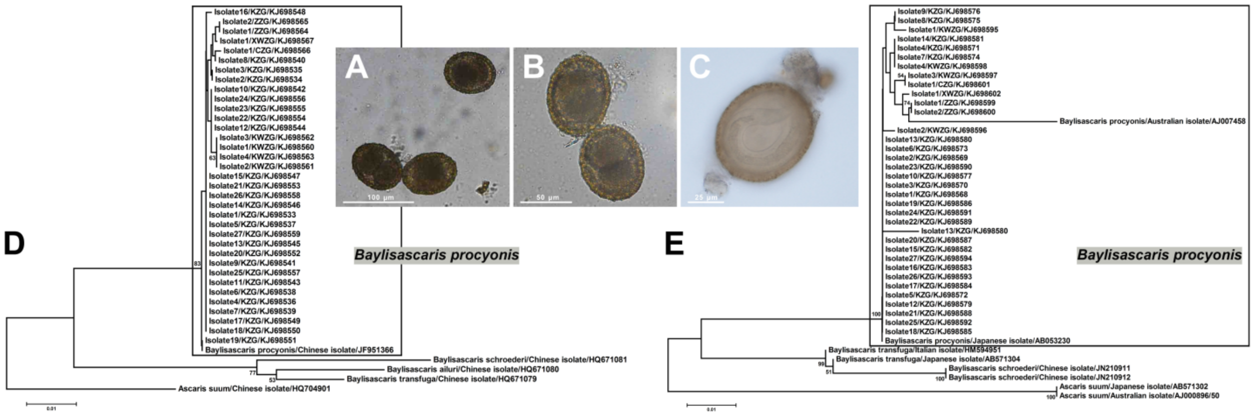

Technical Appendix Figure 2. Morphological and molecular characterization of *Baylisascaris procyonis* parasitic roundworm eggs in captive raccoons in China, 2011–2013. Eggs with 1–2 cells (A and B) or infective larvae (C) detected in the feces of raccoons by the flotation method with NaNO3 were identified as B. procyonis on the basis of their size and morphologic features (*1,2*). Phylogenetic relationships inferred by using GTR + I + G model-based neighbor-joining analysis on the basis of the partial sequences of mitochondrial cox-1 (D) and nuclear ITS-1 (E) from isolates of Baylisascaris species and related ascaridoids. Ascaris suum was used as an outgroup. Neighbor-joining trees were constructed using MEGA 5.0 (www.megasoftware.net). Bootstrapping with 1,000 replicates was performed to calculate the percentage reliability for each node in both genes; only values of ≥50% are shown. Horizontal branch lengths are proportional to genetic distances. Black boxes containing the 35 isolates from this study represent the B. procyonis clusters (sequences are in GenBank: KJ698533–KJ698567 for cox-1 and KJ698568–KJ698602 for ITS-1). Scale bars indicate the number of nucleotide substitutions per site. KZG, Kunming ZG; ZZG, Zhengzhou ZG; XWZG, Xi'an Wildlife ZG; CZG, Chengdu ZG; KWZG, Kunming Wildlife ZG.

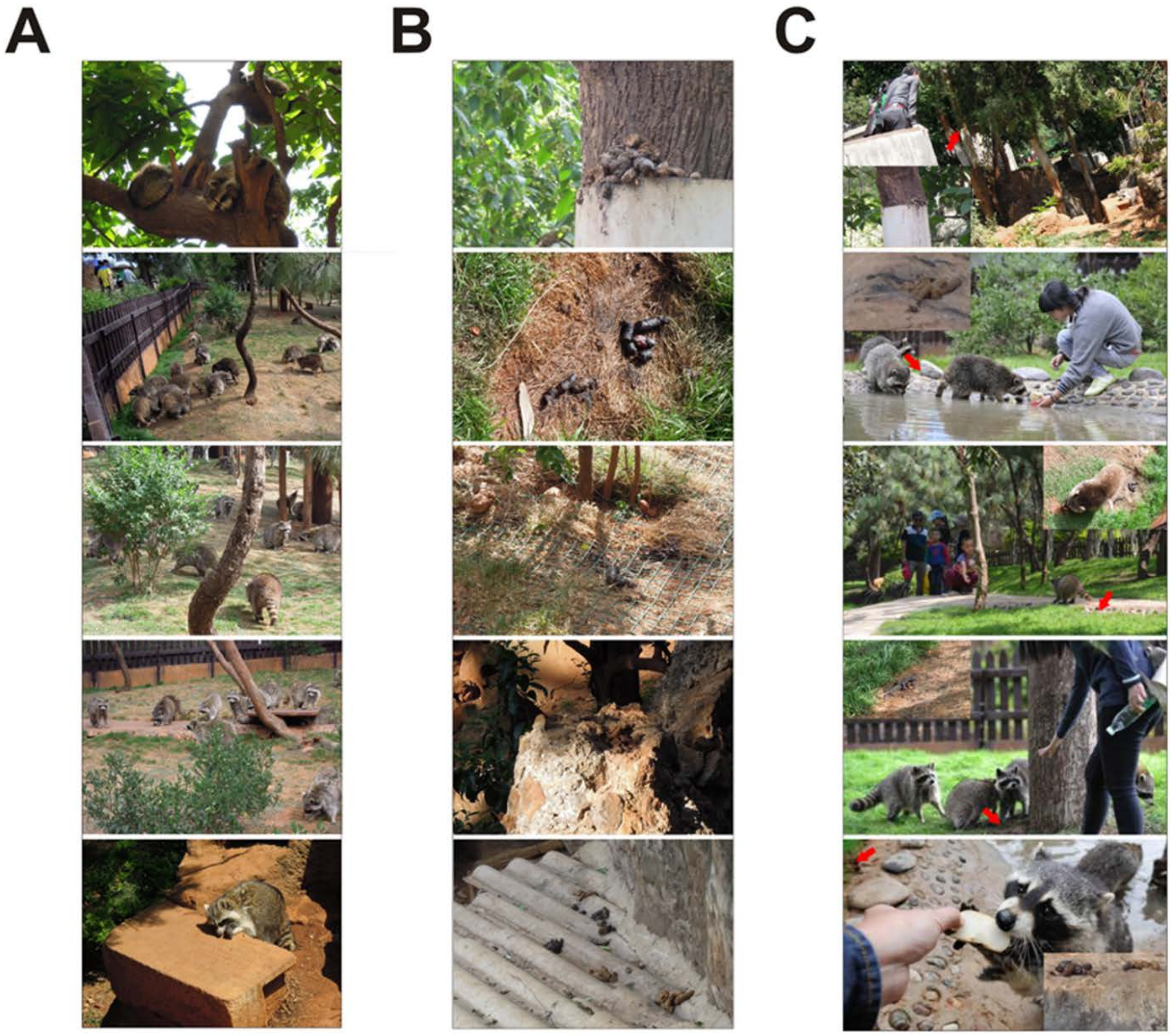

Technical Appendix Figure 3. An image series showing the artificial “niche” created for raccoons in Chinese Zoological Gardens (ZGs) and depicting the potential risk of human infection with *Baylisascaris procyonis* parasitic roundworms. A) Different living environments of raccoons in the studied ZGs. B) Several typical raccoon latrines found in ZGs. C) Human-raccoon interactions, particularly between young children and raccoons. The red arrows within panel C) denote the locations of latrine sites and indicate that these sites are in close proximity to humans and therefore potentially put large numbers of infective *B. procyonis* eggs in the immediate environment of visitors.

References

1. Gavin PJ, Kazacos KR, Shulman ST. Baylisascariasis. Clin Microbiol Rev. 2005;18:703–18. PubMed http://dx.doi.org/10.1128/CMR.18.4.703-718.2005

2. Kazacos K. *Baylisascaris procyonis* and related species. In: Samuel W, Pybus M, Kocan A, editors. Parasitic diseases of wild mammals. 2nd ed. Ames: Iowa State University Press; 2001. p. 301–41.
